# Increased ENaC activity during kidney preservation in Wisconsin solution

**DOI:** 10.1186/s12882-019-1329-7

**Published:** 2019-04-29

**Authors:** Sherif Khedr, Oleg Palygin, Tengis S. Pavlov, Gregory Blass, Vladislav Levchenko, Ammar Alsheikh, Michael W. Brands, Ashraf El-Meanawy, Alexander Staruschenko

**Affiliations:** 1 Department of Physiology, Medical College of Wisconsin, 8701 Watertown Plank Road, Milwaukee, WI 53226 USA; 2 Department of Medicine, Medical College of Wisconsin, Milwaukee, WI 53226 USA; 3 Department of Physiology, Medical College of Georgia, Augusta University, Augusta, GA 30901 USA; 4 Present address: Division of Hypertension and Vascular Research, Henry Ford Hospital, Detroit, MI 48202 USA; 5 Present address: Western Kentucky University, Bowling Green, KY 42101 USA

**Keywords:** ENaC, Wisconsin solution, Kidney transplantation, Shrinkage

## Abstract

**Background:**

The invention of an effective kidney preservation solution capable of prolonging harvested kidney viability is the core of kidney transplantation procedure. Researchers have been working on upgrading the preservation solution quality aiming at prolonging storage time while maintaining utmost organ viability and functionality. For many years, the University of Wisconsin (UW) solution has been considered the gold standard solution for kidney preservation. However, the lifespan of kidney preservation in the UW solution is still limited. Its impact on the epithelial Na^+^ channel (ENaC) activity and its mediated processes is unknown and the primary goal of this study.

**Methods:**

Kidneys harvested from 8 weeks old Sprague Dawley rats were divided into 4 groups depending upon the period of preservation in UW solution. Additional analysis was performed using dogs’ kidneys. ENaC activity was measured using patch clamp technique; protein expression and mRNA transcription were tested through Western blot and RT-qPCR, respectively. A colorimetric LDH level estimation was performed at different time points during UW solution preservation.

**Results:**

Kidney preservation in Wisconsin solution caused reduction of the kidney size and weight and elevation of LDH level. ENaC activity increased in both rat and dog kidneys preserved in the UW solution as assessed by patch clamp analysis. On the contrary, ENaC channel mRNA levels remained unchanged.

**Conclusions:**

ENaC activity is significantly elevated in the kidneys during preservation in UW solution, which might affect the immediate post-implantation allograft function and trajectory post-transplant.

## Background

Renal failure has always been considered a worldwide health debilitating clinical condition. Its incidence has been culminating over the last decade and affecting the quality of life for patients. Renal dialysis can partially replace the filtration function of the kidney but does not compensate for its endocrine, selective re-uptake, and the biosynthetic roles. Dialysis prolongs life but at high cost and significant comorbidities [1–3]. On the other hand, kidney transplantation represents the best alternative that overcomes most of the dialysis disadvantages and has proven to be the most effective, and often the only possible route for treating end-stage renal disease over time [4].

Patients on kidney transplant waiting lists are growing dramatically every year. In the U.S., they have exceeded 100,000 patients while transplantation rates are only at 20% [5]. The current preservation solutions used with kidneys have reduced viability, increase delayed graft function, and higher rate of rejection that becomes more pronounced with preservation time. In fact, most surgeons are very reluctant to transplant a kidney that has been on ice for more than 24 h. This has contributed to the relatively high kidney discards ranging between 5200 and 7300 per year (United Network for Organ Sharing). Improving renal preservation methods to increase organ availability has become  mandatory for covering the gap between wait-listed patients and the limited number of available organs.

The University of Wisconsin (UW) preservation solution has been used for years as the gold standard preservation solution not only for kidneys, but also for other organs, e.g. liver, intestine, and pancreas [6]. It dramatically increased the kidney preservation time up to 72 h [7]. The makeup of UW solution, developed over a 15-year span, is a complex that has been described [8]. However, the importance and role of each component have been controversial. Experiments conducted on UW solution have elucidated the indispensable roles of six components: lactobionic acid, adenosine, hydroxyethyl starch, glutathione, raffinose, and the cations (Na^+^ and K^+^). The specific composition used in UW solution aims to [1] maintain cell integrity and protect it from damage by reactive oxygen species (especially during reperfusion), [2] supply the organ a substrate for energy production to maintain their metabolism, and [3] prevent hypothermic cellular swelling that eventually leads to cell damage.

Even though UW solution has been successful, sparse information is known about its effect on ion channel transporters especially ENaC channels that are abundantly expressed in the distal convoluted tubules and collecting ducts of the kidney [9]. The pivotal role of these channels is well established in controlling total body salt and water homeostasis, and blood pressure [10, 11]. Disturbance in ENaC function has been attributed to the pathophysiology of many sodium handling disorders e.g. Liddle syndrome and pseudohypoaldosteronism type 1 [12] with changes in its activity resulting in blood pressure abnormalities (both hypertension and hypotension) and various kidney diseases. The scientific premise behind our work is to investigate the underlying mechanism(s) affecting the behavior of ENaC and its impact on kidney function and viability during hypothermic storage which could continue and impose a trajectory effect on the graft function after the transplantation. Studying ENaC in this new environment represents a tempting goal that may lead to future potential structural and compositional improvement of the UW solution that may prolong the renal preservation time and/or expand the organ availability.

## Methods

### Animals

Eight-week-old male Sprague Dawley (SD) rats were purchased from Charles River Laboratories (Wilmington, MA). Animal use and welfare adhered to the NIH Guide for the Care and Use of Laboratory Animals following a protocol reviewed and approved by the IACUC at the Medical College of Wisconsin. Rats were kept on a normal rodent diet containing 0.4% NaCl with water supplied ad libitum on a 12-h/12-h light/dark cycle. Animals were randomly allocated to the different experimental groups. Anesthesia was induced using 5% isoflurane gas inhalation, delivered to a transparent and padded chamber housing the animal, via a precision vaporizer and compressed oxygen. Once the animal was unconscious, it was transferred onto a temperature controlled surgical table equipped with a nose cone that supplied continuous delivery of anesthetic. For each animal, both kidneys were flushed with UW solution to clear kidneys of blood and mimic the procedure conducted in the clinic. UW solution was obtained from Bridge to Life Ltd. (Columbia, SC) (Table 1).

**Table 1:** Ingredients of UW solution

INGREDIENT	g/L
Hydroxyethyl starch (Pentafraction)	50.0
Lactobionic acid (as Lactone)	35.83
Potassium dihydrogen phosphate	3.4
Magnesium sulfate heptahydrate	1.23
Raffinose pentahydrate	17.83
Adenosine	1.34
Allopurinol	0.136
Total Glutathione	0.922
Potassium hydroxide	5.61
Sodium hydroxide/Hydrochloric acid (adjust to pH 7.4)	

Osmolarity of UW solution was 320 mOsm, sodium and potassium concentrations were 29 and 125 mEq/L, respectively. After flushing the kidneys, left kidneys were harvested and stored in 50 ml of UW solution for different time points (0, 24, 48, and 72 h) at 4 °C in a cold room. Afterward, kidneys were snap frozen in liquid nitrogen and stored at − 80 °C. The rats were euthanized by thoracotomy under anesthesia.

For experiments with dog kidneys, studies were conducted in conditioned normal male mongrel dogs weighing ~ 25 kg as previously described [13], and all of the experimental protocols were approved by the IACUC at Medical College of Georgia, Augusta University. Freshly isolated kidneys were stored in UW solution and shipped overnight to Medical College of Wisconsin for following electrophysiological and immunohistochemical analyses.

### Western blotting and RT-PCR analysis

For Western blot analysis, kidney cortical lysates were prepared as previously described [14]. Briefly, kidney tissue samples (15–25 mg) were pulse sonicated in Laemmli buffer with a protease inhibitor cocktail (Roche) to a final concentration of 25 mg/ml. The resulting supernatant was subjected to SDS-PAGE, transferred onto nitrocellulose membrane (Millipore) and probed with antibodies for α-, β-, and γ-ENaC subunits (1:1000, Stressmarq Biosciences Inc., Vancouver, cat# SPC-403D, SPC-404D, SPC-405D, respectively), and subsequently visualized by enhanced chemiluminescence.

For RT-PCR analysis, 2 μg of total RNA (determined by Nanodrop 2000; ThermoFisher) was extracted and purified from homogenized kidney using TRIzol reagent (Invitrogen, according to the manufacturer’s protocol) and was used as a template for cDNA formation using random hexamer primer and reverse transcriptase in Thermo Scientific RevertAid First Strand cDNA Synthesis Kit. RT-PCR reactions were carried out on a QuantStudio 6 Flex using Bullseye EvaGreen qPCR Master Mix (MedSci, Valley Park, MO) according to manufacturer’s directions in 10 μL final volume with samples ran in triplicate. Forward and backward primers were designed from the rat sequences for Scnn1a, Scnn1b, Scnn1g, and β-actin. Final Ct values were determined using QuantStudio 6 Flex implemented software, and then α-, β-, and γ-ENaC subunit mRNA copy number were normalized to housekeeping gene β-actin and presented as fold increase compared to RNA isolated from the control group.

### Lactate dehydrogenase (LDH) concentration measurements in UW solution after preservation

A calorimetric lactic acid dehydrogenase (LDH) test was done according to the manufacturer instructions provided with Pierce LDH Cytotoxicity Assay Kit (ThermoScientific). Measurement of the absorbance at 490 nm and 680 nm was done using Gen5 microplate reader.

### Histological and immunohistochemical analysis

Extracted kidneys were prepared for following analysis using 10% formalin solution as described previously [15]. The kidney sections were cut, dried and deparaffinized for subsequent streptavidin-biotin immunohistochemical analysis. For morphological analysis, the kidney tissue samples were stained with haematoxylin-eosin. For immunohistochemical staining, dog tissue sections were incubated for 30 min with anti-β-ENaC antibodies.

### Patch-clamp analysis

Patch clamp analysis was used to assess ENaC activity in freshly isolated, split-opened cortical collecting duct (CCD) tubules. CCDs were isolated from rat kidney cortex as described previously [14–16]. A similar approach was used for isolation of CCDs from dog’s kidneys. Briefly, kidneys were cut into thin slices (< 1 mm) and then placed into ice-cold PBS. The tubules were first mechanically isolated and then split opened with sharpened micropipettes controlled with micromanipulators to gain access to the apical membrane. Single-channel recordings were acquired with Axopatch 200B amplifier (Molecular Devices) interfaced via a Digidata 1440A to a PC running the pClamp 10.2 and subsequently analyzed with Clampex 10.2 software as described [17]. Currents were filtered with low pass Bessel filter LPF-8 (Warner Instruments) at 0.3 kHz. A typical bath solution was used (in mM): 150 NaCl, 1 CaCl_2_, 2 MgCl_2_, 10 HEPES (pH 7.4). The pipette solution for the cell-attached configuration was (in mM): 140 LiCl, 2 MgCl_2_ and 10 HEPES (pH 7.4). The open probability (*P*_*o*_), was used to measure the channel activity within a patch.

### Statistics

Results are presented as mean ± SEM. Data were compared using t-test as well as one-way analysis of variance (ANOVA) with Tukey correction and *P* < 0.05 was considered significant.

## Results

**Fig. 1:**
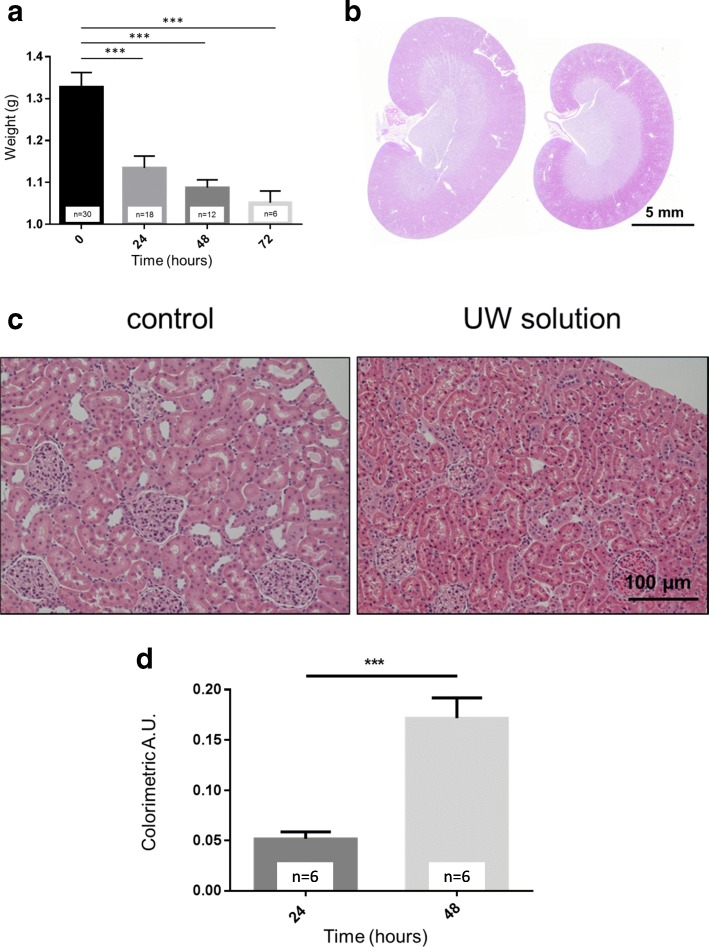
Effect of preservation in UW solution on kidney weight, morphology and LDH levels. **a** Changes in the kidney weight during the different time periods of preservation in UW solution. **b** Kidney morphology after UW solution. The kidney collected from SD rat stored for 48 h in UW solution (right) compared to control kidney (left). **c** Representative histological images (20X) of renal cortex reflecting kidney volume of control kidney and following preservation in UW solution for 48 h. Scale bars are shown. **d** Effect of preservation in UW solution for different time periods on kidney damage as assessed by LDH concentration. Data represent mean ± SEM. *** – *p* < 0.001 compared with control kidneys by one-way ANOVA using Tukey’s multiple comparison post-test. The number of kidneys in each group is shown (one kidney per rat was used for experiments)

Eight-week-old Sprague Dawley (SD) rats were used to test the effects of UW solution. The kidneys were isolated and immediately perfused with UW solution. After isolation, the kidneys were weighed and stored in UW solution for 24, 48, and 72 h, respectively. 24 h of hypothermic static preservation in UW solution resulted in the dramatic reduction of the kidney size and weight compared to the control group (i.e., freshly isolated, flushed but not preserved in UW solution kidneys (0 h)). Hypothermic storage in UW solution for 24, 48, and 72 h resulted in kidney shrinkage and a significant reduction in their weights by 16, 18, and 21%, respectively (Fig. 1a and b). As shown on histological images in Fig. 1c, the reduction in the kidney weight was due to kidney shrinkage and a decrease in the size of both glomeruli and tubules. Importantly, the kidney cortex was specifically smaller and dense in the preserved kidney. Thus, the luminal space in the collecting ducts was notably reduced after incubation in UW solution.

LDH release by damaged tissues is widely used in clinics as a marker of cell death. Subsequently, in order to confirm and monitor renal cell structural integrity over time in the preservation solution, LDH concentration measurements were conducted during storage in UW solution for 24 and 48 h. Results showed increased time-dependent renal damage underscored by the rising LDH level detected in the preservation solution (Fig. 1d).

**Fig. 2:**
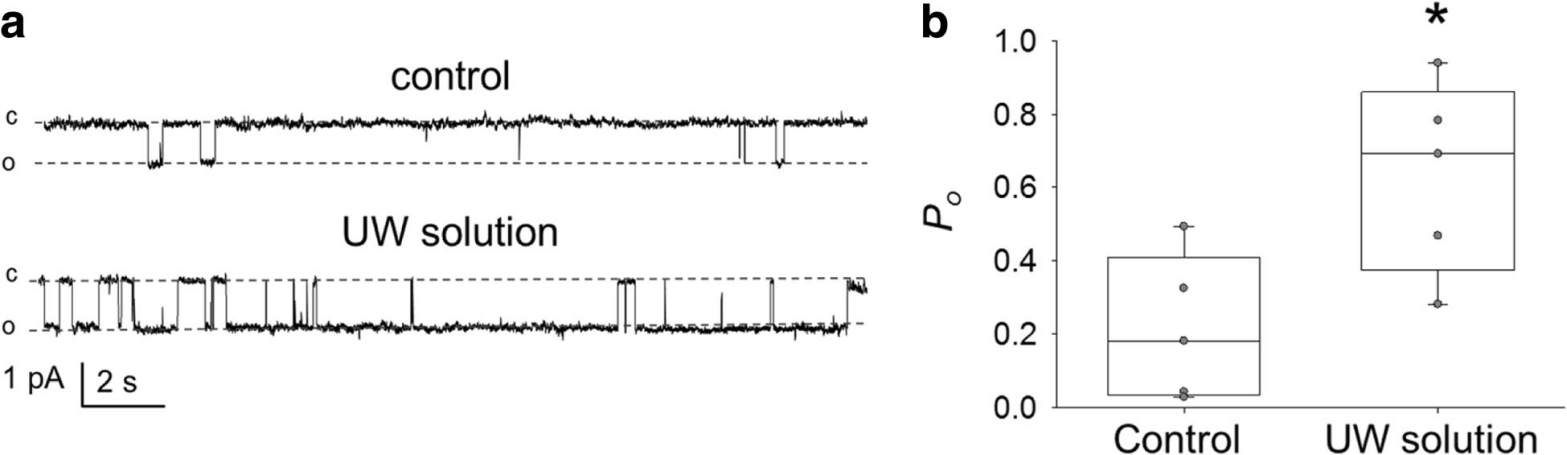
ENaC activity in isolated cortical collecting duct (CCD) tubules. **a** Representative current traces from cell-attached patches containing ENaC and recorded from the apical membrane of split-open CCD cells. The kidneys freshly isolated either from Sprague-Dawley rats were used as control or following 24 h of preservation in UW solution. Holding potential is − 40 mV. “c” and “o” represent closed and open states. **b** Summary graphs of ENaC open probability (*P*_*o*_). * *p* < 0.05, *n* = 5 in each group (4 and 3 rats in control and UW groups, respectively)

**Fig. 3:**
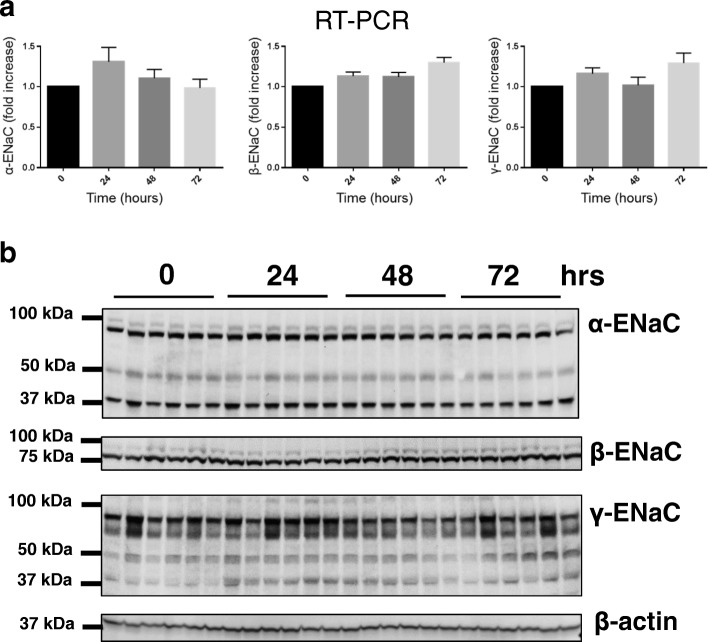
The expression of ENaC subunits after preservation in UW solution. **a** RT-PCR analysis of mRNA expression of α-, β-, and γ-ENaC subunits after cold static preservation in UW solution for different time points, *n* = 6 kidneys (1 kidney/rat) in each group. **b** Western blot analysis of ENaC expression in kidney total lysates isolated from SD rats after cold static kidney preservation in UW solution for 0, 24, 48, and 72 h. Each lane represents one kidney

ENaC activity depends on cell volume and could be altered due to osmotic balance, shrinkage, flow and other mechanical stimuli. The effect of renal preservation in UW solution on ENaC channel activity and expression and the subsequent influence on renal integrity has not been previously addressed to our knowledge. Therefore, we tested if kidney preservation in UW solution results in modulation of ENaC activity and expression. To determine any potential changes in ENaC activity, patch clamp analysis of channel activity in split-open tubules isolated from control SD rats or rats after UW solution was implemented. We found a marked increase in ENaC activity compared to the control group, documented by the increased channel open probability (*P*_*o*_) (Fig. 2); the total number of channels per patch tested did not vary significantly between the test and the control groups. The following experiments tested whether UW solution alters the RNA transcriptional level and protein expression of all three ENaC subunits. However, our results confirmed electrophysiological analysis that changes in the channel activity were due to increase in *P*_*o*_ and were not associated with changes in any of the three subunits i.e. α, β, and γ subunits neither at the mRNA transcriptional level (Fig. 3a) nor protein expression (Fig. 3b).

**Fig. 4:**
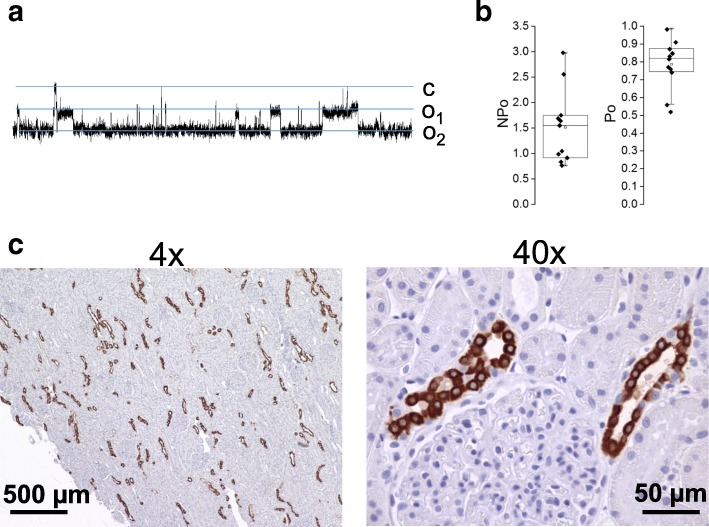
ENaC expression and activity in dog kidney after preservation in UW solution. **a** Current traces from a representative cell-attached patch that contained ENaC. This patch was formed on the apical membrane of a principal cell in an isolated split-open collecting duct that was isolated from dog kidney. The holding membrane potential is − 60 mV. “c” and “o” denote closed and open current levels, respectively. **b** Summary graph of ENaC activity (*NP*_*o*_) and channel open probability (*P*_*o*_) indicates high channel activity in dog kidneys preserved in UW solution for 24 h. **c** Representative images of immunohistochemical staining of β-ENaC (shown in brown) in dog kidney cortical sections

We also tested ENaC activity and expression in the dog kidneys preserved approximately 24 h in UW solution. Shown in Fig. 4a is a representative activity of ENaC measured in freshly isolated split open dog CCD. As summarized in Fig. 4b, both ENaC activity (*NP*_*o*_) and *P*_*o*_ were very high (*P*_*o*_ was 0.78 ± 0.04, *N* = 11) following storage of the dog kidneys in UW solution. Typically, the activity of endogenous ENaC in mammalian collecting ducts isolated from animals in normal conditions is significantly lower [18]. Shown in Fig. 4c are images from a kidney immunohistochemically stained for β-ENaC (shown in brown) at 4x and 40x. Negative control (stained with secondary antibodies in the absence of primary antibodies) did not show any staining (data not shown).

## Discussion

Hypothermic cell swelling has been blamed, in part, for the cellular injury during kidney preservation. The main role of extracellular sodium is to counteract the osmotic pressure caused by the intracellular non-diffusible osmols, thus keeping the cell volume regulated and constant. Hypothermic cellular swelling has been proven to have a detrimental impact on kidney performance after transplantation as cellular, and interstitial edema cause tissue vascular bed compression and occlusion, resulting in poor post-transplantation prognosis [19].

Addition of a colloidal substance (e.g., hydroxyethyl starch in UW solution), as well as non-permeates compounds (lactobionate and raffinose), has improved cellular and interstitial edema [20]. Therefore, maintaining cellular volume and composition is the ultimate goal behind the specific UW solution composition. However, the current study, as well as previous reports by others, documented a reduction in kidney weight after hypothermic static preservation in UW solution [21–24]. The aforementioned reduction in kidney weight after preservation in UW has been also observed in other species. For instance, studies by Schreinemachers et al. found similar effects of UW solution on the porcine kidney [22]. Although UW solution is isosmotic, the detrimental weight reduction could be abrogated by incorporation of colloid in UW solution [23].

The impact of kidney shrinkage on renal function is still unclear. However, previous work conducted in other laboratories has found that cell shrinkage was accompanied by cellular apoptosis and damage [25–27]. It has been shown that ENaC channels are mechanosensitive, and they can be activated by a vast variety of mechanical stimuli e.g., shear stress and flow stimulation [28–30], hydrostatic pressure, cell swelling [31], and cell shrinkage [32]. In agreement with these data, we observed increased ENaC channel activity, determined by increased *P*_*o*_ after preservation in UW solution. RT-PCR and Western blot analyses showed stability in different ENaC subunit mRNA and protein levels over different preservation time intervals tested.

To confirm our observations, we addressed ENaC activity using another animal species, which we believe enhances the translational ramifications of our studies. The dog represents an established model for renal studies, which is more similar to humans than any rodent model. In agreement to our previous observations conducted on SD rats, measurement of ENaC activity in dog kidney preserved in UW solution revealed enhanced ENaC *P*_*o*_ which suggests a more universal effect of UW solution than a species-specific effect. Interestingly, a previous study on a lung transplant model revealed that ENaC mRNA and protein levels were decreased in transplanted lungs [33]. Augmentation in ENaC *P*_*o*_ could be attributed to the mechanical stimulation (i.e. shrinkage), and/or a response to one or more of the components of the preservation solution. This increase in ENaC activity might be a part of a cellular compensatory mechanism that is called regulatory volume increase [32, 34, 35] that tries to increase solute, and thus water influx, aiming at improving the cell volume back to normal. The failure to restore the original cell size may be due to the low sodium concentration in UW solution (30 mEq/l) which could not create a sufficient concentration gradient. In addition, the negative charge of lactobionic acid found in UW solution could hinder this compensatory volume rectifying sodium influx into shrunken cells. Also, large amounts of poly-carbohydrates in starch and raffinose, which is a trisaccharide composed of galactose, glucose, and fructose, can serve as a source of the monosaccharides that are capable of increasing channel activity.

## Conclusions

Current findings emphasize the critical role of ENaC in the maintenance of kidney function when kidneys are preserved in preservation solution. It is not known, and beyond the scope of this manuscript, how long the increased ENaC activity will be maintained after implantation and reperfusion of the transplanted kidney and how much it contributes to delayed graft function seen if kidney is transplanted after prolonged cold preservation time. High ENaC activity can reduce urine flow, increase interstitial sodium concentration and increase kidney interstitial swelling. Interestingly a recent study revealed that albuminuria in kidney transplant recipients was associated with elevated ENaC activity and the urine from such patients was able to activate ENaC currents in vitro [36]. However, the mechanisms of ENaC activation during kidney preservation and following transplantation are most likely independent of each other. Effect of UW solution on other channels and transporters in the kidney is also unknown and should be studied to understand the contributions of renal ion transport during kidney preservation.

## Abbreviations

ENaC: Epithelial sodium channel
LDH: Lactate dehydrogenase
UW: University of Wisconsin Solution
